# Transcription of reference genes used for quantitative RT-PCR in Atlantic salmon is affected by viral infection

**DOI:** 10.1186/1297-9716-42-8

**Published:** 2011-01-18

**Authors:** Marie Løvoll, Lars Austbø, Jorunn B Jørgensen, Espen Rimstad, Petter Frost

**Affiliations:** 1 Section for immunoprophylaxis, National Veterinary Institute, P.O. Box 750 Sentrum, 0106 Oslo, Norway; 2 Department of Basic Sciences and Aquatic Medicine, Norwegian School of Veterinary Science, P.O. Box 8146 Dep, 0033 Oslo, Norway; 3 Norwegian College of Fishery Science, University of Tromsø, 9037 Tromsø, Norway; 4 Department of Food Safety and Infection Biology, Norwegian School of Veterinary Science, P.O. Box 8146 Dep, 0033 Oslo, Norway; 5 Intervet Norbio AS, Thormøhlensgate 55, 5008 Bergen, Norway

## Abstract

Relative quantification using RT-qPCR is a widely used method for transcription profiling. Transcript levels of target genes in fish after experimental infection is often reported without documentation of stably transcribed reference genes. We present results demonstrating that transcription of typically used reference genes in Atlantic salmon is not stable during experimental infection with salmon pancreas disease virus (SPDV). Transcript levels 0 to 6 weeks after challenge revealed statistically significant changes between time-points that corresponded with a peak in viral load 3 weeks after challenge. The results emphasize the need for thorough method validation prior to transcriptional studies during viral infections.

## Introduction, Methods and Results

For transcription profiling of a limited number of genes, relative quantification using RT-qPCR is a widely used method. Levels of target gene transcripts are estimated relative to a reference gene shown to be evenly transcribed in the relevant tissues. Several publications report transcription studies using reference genes without proper validation of their stability. Only a few years ago, ribosomal RNA was widely accepted as an internal control suitable for normalisation of most RT-qPCR data. This assumption has however changed into a general understanding that the transcript levels of reference genes may vary considerably [1].

Compared to inbred mammalian species, study of gene transcription in salmonid fish is complicated by high variation between individuals and multiple subtypes and isoforms of genes [2]. Furthermore, transcription studies in fish are often comprehensive and designed to follow a population of healthy fish during experimental challenge. To limit otherwise labour intensive and expensive studies, validation of reference genes are typically done on naïve fish omitting statistical analyses. The results are often considered to be valid for experimentally treated fish, even though for instance a virus infection has the potential to change the transcription of cellular genes to facilitate virus production. In contrast to the large number of articles describing target gene transcription after infection in fish, only a limited number of articles demonstrate reference gene validation in different tissues and cells isolated from salmon before, during and after viral infection [3-6].

In the present study we performed a cohabitant challenge of Atlantic salmon (*S. salar*) parr/fingerlings with salmon pancreas disease virus (SPDV). Cohabitation represents a natural waterborne route of pathogen exposure [7], and should stimulate natural anti SPDV defence mechanisms, including important innate and acquired immune responses [8]. The transcript levels of four commonly used reference genes; elongation factor 1αB (EF1αB), 18S rRNA, β-actin and structural ribosomal protein S20 (RPS20), were quantified in tissue from five fish 0, 1, 3, and 6 weeks after challenge. Samples from organs known to be involved in defence and clearance of viral infections in fish, including head kidney, spleen, gill and intestine were examined [9]. Heart was included as it is known to be a target organ of SPDV multiplication and pancreas disease pathology [10]. Serum samples were obtained for virus prevalence studies.

Unvaccinated Atlantic salmon (*S. salar*) (~30 g), were obtained from SalmoBreed (Matre, Norway). 62 fish were kept in 150 L fresh water at 10 to 13°C, with an oxygen saturation > 65%. On day 0, 12 fish were i.p. injected with 200 μL of a SPDV isolate (SAV-3) [11] propagated to second passage in CHSE-214 cell culture. The shedders were placed back into the tank with 50 cohabitees. All fish were anaesthetized by metacainum (0.1 mg/mL) bath treatment for 2 to 3 min before handling. Tissue samples were stored in RNA*later *(Ambion, Applied Biosystems, Foster City, CA, USA) at 4°C and total RNA was isolated using the RNeasy Mini Kit (Qiagen, Hilden, Germany). To eliminate any genomic DNA contamination, RNA was treated with DNase (TURBO DNA-*free*™, Ambion) prior to cDNA synthesis. Viral RNA was extracted from serum samples using QIAamp Viral RNA Mini kit (Qiagen). The experiment was approved by the National Committee of Ethics as required by Norwegian law.

The RT-qPCR assays were performed in duplicate with a Stratagene (Stratagene, La Jolla, CA, USA) detection system at the following conditions: 48°C/30 min, 95°C/10 min, 40 cycles of 95°C/15 sec and 60°C/60 sec. Data were captured using Stratagene MxPro Mx3005P QPCR software. Each reaction contained 2× Master Mix, 40× MultiScribe Reverse Transcriptase and RNase Inhibitor Mix, primers (900 nM), TaqMan^® ^MGB probe (250 nM), 25 ng RNA (2.5 ng for 18S rRNA), and nuclease free water to a final volume of 25 μL. Primer and probe sequences were obtained/redesigned from Olsvik et al. [12] (Table 1). To verify specific priming, each primer set was tested using SYBR Green followed by generation of dissociation curves [13]. Two-fold serial dilutions of total RNA from spleen (200 to 1.5 ng for EF1αB, β-actin and RPS20; 20 to 0.15 ng for 18S rRNA) were made for efficiency (*E*) calculations according to the equation *E *= 10^(1/-slope)^. All assays were found to be quantitative within the range 1.5 to 100 ng RNA (0.15 to 10 ng for 18S rRNA) demonstrating efficiencies close to 2 (Table 1). The primers and probe used for detection of viral RNA (Table 1) were obtained from Hodneland et al. and each reaction was run in triplicate under conditions described earlier [14].

**Table 1:** Oligonucleotide sequences, amplicon lengths, GenBank accession numbers and standard curve evaluation for qPCR assays.

Gene	Oligonucleotide sequence (5'-3')	Amplicon (bp)	GenBank acc. no	Slope	**R**^**2**^	Efficiency (*E*)*
EF1αB	TGCCCCTCCAGGATGTCTAC	57	BG933897	3.460	0.979	1.94
	CACGGCCCACAGGTACTG					
	6FAM-AAATCGGCGGTATTGG-MGBNFQ					
RPS20	GCAGACCTTATCCGTGGAGCTA	85	BG93667	3.055	0.979	2.12
	TGGTGATGCGCAGAGTCTTG					
	6FAM-CCTCAAGGTGAAGGGA-MGBNFQ					
β-actin	CCAAAGCCAACAGGGAGAAG	91	BG933897	3.173	0.996	2.06
	AGGGACAACACTGCCTGGAT					
	6FAM-TGACCCAGATCATGTTT-MGBNFQ					
18S rRNA	CCCCGTAATTGGAATGAGTACACTTT	98	AJ427629	3.092	0.978	2.10
	ACGCTATTGGAGCTGGAATTACC					
	6FAM-CTTTAACGAGGATCCATTGG-MGBNFQ					
SPDV	CCGGCCCTGAACCAGTT	107	AY604235			
	GTAGCCAAGTGGGAGAAAGCT					
	6FAM-CTGGCCACCACTTCGA-MGBNFQ					

* The amplification efficiency of each primer set was assessed according to the equation *E *= 10^(1/-slope)^.

**Figure 1:**
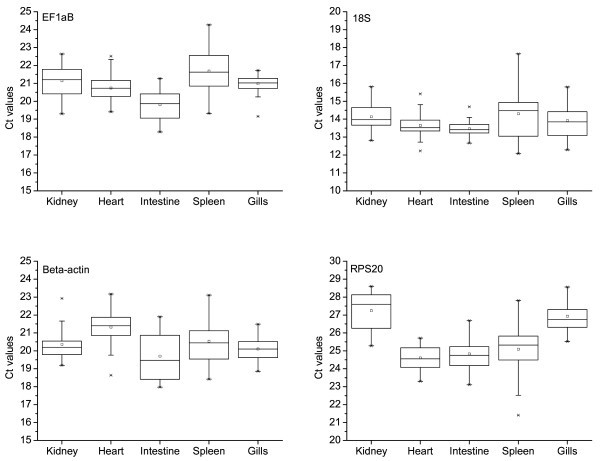
**Tissue related transcript levels of potential reference genes from 0 to 6 weeks after challenge with SPDV**. The raw cycle threshold (Ct) data of each reference gene in all samples (*n *= 20) are represented in a box-and-whisker diagram. Boxes represent the 25^th ^and 75^th ^percentiles with medians indicated; whiskers represent the highest and lowest values. Mean values are indicated by a square. The 1^st ^and 99^th ^percentiles are indicated by X below and above each box, respectively.

Transcription levels of the candidate reference genes in heart, head kidney, spleen, intestine and gills from five fish were calculated 0, 1, 3 and 6 wpc. Data from each sampling (*n *= 5) were included in the total data set across time-points (*n *= 20). The biological variation (individual variation and variation in infectious status) at all time-points and the technical variation introduced were summarized for each tissue (Figure 1). The levels of 18S rRNA showed the lowest variation. The Ct span varied from 1.4 cycles in intestine to 4.9 cycles in spleen (*n *= 20) (Table 2). However, 18S rRNA necessitates high transcription levels of the target gene in order to use one RNA dilution for both target gene and 18S rRNA without entering the non-linear phase of amplification in either direction. EF1αB was transcribed at a lower level than 18S rRNA, closer to the levels of typical target genes. The Ct values detected for EF1αB spanned from 2.3 to 2.9 cycles in all tissues except the spleen. This indicated that a 4.9- (gills), 6.9- (head kidney and intestine) and a 7.4- (heart) fold change in target gene transcription must be achieved for the change to be significant using EF1αB as reference gene (Table 2). The highest variation in EF1αB transcript levels was detected in the spleen, where a 29.8-fold change in target gene transcription would be required to normalise RT-qPCR data. Transcription of β-actin and RPS20 generally showed higher variation than EF1αB, except from in the gills and heart, respectively (Table 2).

**Table 2:** Transcription stability of reference genes illustrated by the Ct ranges obtained from each tissue throughout 6 weeks post SPDV challenge (*n *= 20).

Gene	Ct range (fold change)				
	Head kidney	Heart	Intestine	Spleen	Gills
EF1αB	19.6-22.4 (6.9X)	19.5-22.4 (7.4X)	18.4-21.2 (6.9X)	19.3-24.2 (29.8X)	19.3-21.6 (4.9X)
18S rRNA	12.9-15.3 (5.2X)	12.5-14.9 (5.2X)	12.7-14.1 (2.6X)	12.4-17.3 (29.8X)	12.5-15.6 (8.5X)
β-actin	19.4-22.4 (8.0X)	18.8-22.8 (16.0X)	18.0-21.4 (10.5X)	18.5-23.0 (22.6X)	19.2-21.3 (4.2X)
RPS20	25.2-28.4 (9.1X)	23.3-25.5 (4.5X)	23.2-26.6 (10.5X)	21.4-27.7 (78.7X)	25.7-28.5 (6.9X)

Level of target gene transcription regulation (fold change) that can be significantly identified using the respective reference genes is shown in brackets.

**Figure 2:**
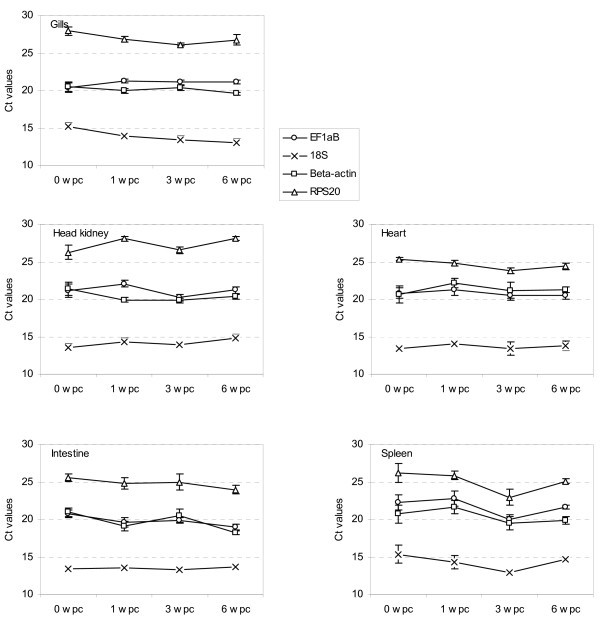
**Transcription profiles of reference gene candidates after experimental challenge of Atlantic salmon with SPDV**. Transcription of EF1αB, 18S rRNA, β-actin and RPS20 0 to 6 weeks post challenge was quantified by RT-qPCR. Data are expressed as raw cycle threshold (Ct) values and represented as mean ± SD (*n *= 5). Wpc = weeks post challenge.

Cohabitant challenge models represent a natural water borne route of pathogen exposure, but also complicate the final analyses as fish sampled at each time-point may be at different stages of infection. Analysis of individual gene transcription levels throughout infection revealed significant changes between time-points (*p *< 0.05) of all genes except β-actin in the spleen, 18S rRNA in the intestine and heart, and EF1αB in the heart (ANOVA) (Figure 2). It should be emphasized that the number of fish analysed per sampling was low (*n *= 5), especially considering the cohabitant challenge model applied. This probably resulted in an artificially low intragroup variation. No virus RNA was detected in serum samples from 0 and 1 wpc. The viral load peaked 3 wpc, however, only 7 out of 10 animals were positive. At 6 wpc, 9 out of 10 animals were positive.

## Discussion

Experimental challenge trials are often designed to study the immunological responses after infection. Spleen and head kidney are primary lymphoid organs in fish, and are typically sampled for immune gene transcription studies. The presence of highly activated immune cells in these organs after infection obviously affects the transcript levels of a number of genes. The present study shows that transcription of typical reference genes significantly changed during infection, emphasizing the need for thorough method validation prior to transcriptional studies.

Although caution must be taken not to over-interpret the results from the individual time-points, there seemed to be an increase in transcription of all candidate genes in the spleen 3 wpc. This coincided in time with the peak of virus production. The results imply that variation in transcription occurs between time-points as a result of the ongoing viral infection. The serum viral load likely mirrors the production of viral particles in the fish, and it has recently been shown that shedding of SPDV from the fish population into the surrounding water coincides with viremia [15].

The effect of SPDV replication on the synthesis of cellular macromolecules has not been studied in detail, but the replication of alphaviruses in mammalia has been shown to inhibit transcription and shut down protein synthesis [16]. Therefore, it could be assumed that high SPDV replication would reduce transcription in general. However, the proportion of infected versus non-infected cells in tissue samples is unknown, and the influx of highly biologically active cells of the immune system to infected organs would influence the overall transcription activity in tissues. Infection with spring viremia of carp virus (SVCV) has been shown to influence transcription of ribosomal genes in the common carp (*Cyprinus carpio*) [17], and it is likely to assume a similar effect of other viral infections in other species. The complex interactions between viral proteins and immunological signal cascades may influence the transcription of alleged stably transcribed reference genes.

In conclusion, we report the transcription variation of four reference genes commonly used for normalisation of RT-qPCR data in five tissues from Atlantic salmon during experimental SPDV infection. The levels of target gene transcription changes that can be significantly identified in each tissue will be limited by variation in the reference gene transcription likely to result from the viral infection. Among the candidate genes tested, EF1αB emerged as most suitable, although requiring up to 8-fold change in target transcription in most organs to be valid.

## Competing interests

The authors declare that they have no competing interests.

## Authors' contributions

All authors designed the experimental challenge and participated in sampling. ML wrote most of the manuscript and did the experimental analysis together with LA. PF, LA and ER contributed to writing and PF contributed to statistical analysis and data presentation. All authors read and approved the final manuscript.

## References

[B1] Huggett J Dheda K Bustin S Zumla A Real-time RT-PCR normalisation; strategies and considerations Genes Immun 2005 6 279 284 10.1038/sj.gene.6364190 15815687

[B2] Brunet FG Crollius HR Paris M Aury J-M Gibert P Jaillon O Laudet V Robinson-Rechavi M Gene loss and evolutionary rates following whole-genome duplication in teleost fishes Mol Biol Evol 2006 23 1808 1816 10.1093/molbev/msl049 16809621

[B3] Ingerslev H-C Pettersen EF Jakobsen RAa Petersen CB Wergeland HI Expression profiling and validation of reference gene candidates in immune relevant tissues and cells from Atlantic salmon (*Salmo salar *L.) Mol Immunol 2006 43 1194 1201 10.1016/j.molimm.2005.07.009 16139890

[B4] Jorgensen SM Kleveland EJ Grimholt U Gjoen T Validation of reference genes for real-time polymerase chain reaction studies in Atlantic salmon Mar Biotechnol 2006 4 398 408 10.1007/s10126-005-5164-4 16676145

[B5] Julin K Johansen LH Sommer AI Reference genes evaluated for use in infectious pancreatic necrosis virus real-time RT-qPCR assay applied during different stages of an infection J Virol Methods 2009 162 30 39 10.1016/j.jviromet.2009.07.003 19638286

[B6] Mitter K Kotoulas G Magoulas A Mulero V Sepulcre P Figueras A Novoa B Sarropoulou E Evaluation of candidate reference genes for QPCR during ontogenesis and of immune-relevant tissues of European seabass (*Dicentrarchus labrax*) Comp Biochem Physiol B Biochem Mol Biol 2009 153 340 347 10.1016/j.cbpb.2009.04.009 19398033

[B7] Nordmo R Strengths and weaknesses of different challenge methods Dev Biol Stand 1997 90 303 309 9270858

[B8] Aoki M Kondo M Kawai K Oshima S Experimental bath infection with *Flavobacterium psychrophilum*, inducing typical signs of rainbow trout *Oncorhynchus mykiss *fry syndrome Dis Aquat Org 2005 67 73 79 10.3354/dao067073 16385811

[B9] Press CM Evensen O The morphology of the immune system in teleost fishes Fish Shellfish Immun 1999 9 309 318 10.1006/fsim.1998.0181

[B10] Taksdal T Olsen AB Bjerkaas I Hjortaas MJ Dannevig BH Graham DA McLoughlin MF Pancreas disease in farmed Atlantic salmon, *Salmo salar *L., and rainbow trout, *Onchorhynchus mykiss *(Walbaum) in Norway J Fish Dis 2007 30 545 558 10.1111/j.1365-2761.2007.00845.x 17718709

[B11] Christie KE Fyrland K Holtet L Rowley HM Isolation of pancreas disease virus from farmed Atlantic salmon, *Salmo salar *L., in Norway J Fish Dis 1997 21 391 394 10.1046/j.1365-2761.1998.00127.x

[B12] Olsvik PA Lie KK Jordal A-EO Nilsen TO Hordvik I Evaluation of potential reference genes in real-time RT-PCR studies of Atlantic salmon BMC Mol Biol 2005 6 21 29 10.1186/1471-2199-6-21 16293192 PMC1314898

[B13] Ririe KM Rasmussen RP Wittwer CT Product differentiation by analysis of DNA melting curves during the polymerase chain reaction Anal Biochem 1997 245 154 160 10.1006/abio.1996.9916 9056205

[B14] Hodneland K Endresen C Sensitive and specific detection of Salmonid alphavirus using real-time PCR (TaqMan) J Virol Methods 2006 131 184 192 10.1016/j.jviromet.2005.08.012 16202457

[B15] Andersen L Hodneland K Nylund A No influence of oxygen levels on pathogenesis and virus shedding in Salmonid alphavirus (SAV)-challenged Atlantic salmon (*Salmo salar *L.) Virol J 2010 7 198 10.1186/1743-422X-7-198 20727205 PMC2936311

[B16] Schlesinger S Schlesinger MJ Knipe DM, Howley PM Togaviridae; the viruses and their replication Fields' Virology 2001 Philadelphia: Lippincott, Williams and Wilkins 895 916

[B17] Forlenza M de Carvalho Dias JDA Vesely ET Pokorova D Savelkoul HFJ Wiegertjes GF Transcription of signal-3 cytokines, IL-12 and IFNαβ, coincides with the timing of CD8αβ up-regulation during viral infection of common carp (*Cyprinus carpio *L.) Mol Immunol 2008 45 1531 1547 10.1016/j.molimm.2007.10.010 18022233

